# Early prediction of metastatic insulinoma using tumor size and biochemical markers: a retrospective cohort study

**DOI:** 10.3389/fendo.2026.1905732

**Published:** 2026-08-25

**Authors:** Annie Mathew, Ben Freudenberg, Marc Wichert, Frank Weber, Benedikt M. Schaarschmidt, Johanna Braegelmann, David Kersting, Dagmar Fuhrer, Harald Lahner

**Affiliations:** 1 Department of Endocrinology, Diabetes and Metabolism and Division of Laboratory Research, University Hospital Essen, Endocrine Tumor Center at WTZ/Comprehensive Cancer Center and ENETS Center of Excellence, University of Duisburg-Essen, Essen, Germany; 2 Center of Endocrine Medicine, University of Duisburg-Essen, Essen, Germany; 3 Central Laboratory, University Hospital Essen, University Duisburg-Essen, Essen, Germany; 4 Department of General, Visceral and Transplantation Surgery, Section of Endocrine Surgery, University Hospital Essen, University Duisburg-Essen, Essen, Germany; 5 Institute for Diagnostics and Interventional Radiology and Neuroradiology, University Hospital Essen, University of Duisburg-Essen, Essen, Germany; 6 Department of Nuclear Medicine, West German Cancer Center, German Cancer Consortium (DKTK) and National Center for Tumor Diseases site, University Hospital Essen, University of Duisburg-Essen, Essen, Germany

**Keywords:** functioning pancreatic NET, hypoglycemia, metastatic insulinoma, neuroendocrine tumors, risk prediction model

## Abstract

**Introduction:**

Insulinomas are rare functional pancreatic neuroendocrine tumors. Metastasis is found in up to 15% of patients and is difficult to predict at diagnosis.

**Methods:**

We retrospectively analyzed 64 patients (56.2% female, median age 49 years) with biochemically confirmed insulinoma treated at a European Neuroendocrine Tumor Society Center of Excellence between 2010 and 2024, assessing clinical characteristics, tumor biology, diagnostics, treatments and survival (median follow-up 23 months) .

**Results:**

Metastasis was present in 27% of cases (17/64). Metastatic insulinomas had significantly larger primary tumors (median 32 vs. 15 mm, p < 0.001), higher Ki-67 indices (median 11% vs. 2%, p < 0.001), shorter overall survival (median 51 months vs. not reached, p < 0.001) and a shorter interval from symptom onset to diagnosis (median 2 vs. 8 months, p < 0.05). Surgery was performed in 79% of patients (39/45 non-metastatic vs. 10/17 metastatic insulinomas). All metastatic cases required further multimodal therapy, including pharmacological and local-ablative treatments. A logistic prediction model based on tumor size, fasting glucose and the insulin-to-C-peptide ratio was developed to predict metastasis in insulinoma patients and showed excellent performance (AUC 0.96, sensitivity 90.9%, specificity 93.8%) in the insulinoma cohort. ROC analysis identified clinically relevant size thresholds, with a tumor size cut-off at 19 mm capturing all metastatic insulinomas with available size data and a cut-off at 24 mm improving specificity for metastasis to 84.6%. Applied to 24 published insulinoma cases, the model achieved a classification accuracy of 75%. Stratification into three risk categories (“low,” “medium,” and “high”) further enhanced clinical interpretability. In line with molecular studies, metastatic insulinomas in our cohort exhibited disproportionate proinsulin secretion in the available cases, supporting the concept of β-cell dedifferentiation with impaired prohormone processing as a feature of aggressive disease.

**Discussion:**

Our analysis highlights relevant clinical and biological distinctions between non-metastatic and metastatic insulinomas. Based on three diagnostic parameters, we propose a predictive model for early risk stratification to guide staging intensity and follow-up in insulinoma management.

## Introduction

Insulinomas are the most common functional pancreatic neuroendocrine tumors (pNETs), yet they remain rare, with an estimated incidence of only 1–4 cases per million people annually (1, 2). Insulinomas arise from pancreatic beta cells and are defined by an inadequate secretion of insulin causing severe hypoglycemia. The diagnosis is based on the presence of Whipple’s triad which includes symptoms of hypoglycemia in combination with documented low plasma glucose concentrations and relief of symptoms following glucose administration alongside inappropriately elevated levels of insulin and C-peptide during hypoglycemia (1).

While most insulinomas are non-metastatic and can be cured by surgery, 10–15% of cases exhibit aggressive (“malignant”) tumor behavior (3). They form metastases locally or in distant organs. Patients with metastatic insulinoma have a significantly shorter survival time (1). However, at the time of diagnosis, non-metastatic and aggressive insulinomas often appear clinically and biochemically indistinguishable (2). This is consistent with the established biology of pNETs. All pNETs possess an inherent potential for aggressive transformation driven by the progressive accumulation of proliferative and molecular alterations. Although immunohistochemical and genetic changes of an insulinoma have been found to correlate with metastatic and non-metastatic disease courses, they have primarily been studied in retrospective case series and are not widely available in routine clinical diagnostics (3). These observations highlight the need for robust early risk-stratification strategies capable of detecting this transition prior to the development of metastatic disease. Unlike prior studies focusing on the diagnosis of insulinoma (4), predictive cut-offs for distinguishing aggressive from non-metastatic insulinomas are rarely described (5). In clinical practice, this distinction is essential for risk stratification, treatment planning and prognosis.

Our study addresses this unmet need by analyzing a well-defined, single-center longitudinal cohort of 64 patients with biochemically confirmed insulinoma. All patients were diagnosed and followed under a standardized diagnostic and therapeutic protocol, including structured dynamic endocrine testing, multidisciplinary tumor board discussion and long-term follow-up. We evaluate differences in clinical presentation, tumor biology, test performance and survival outcomes to propose a predictive model allowing for the early identification of high-risk patients and guiding personalized management strategies in this rare but impactful disease.

## Materials and methods

Patients were identified from our NET database at the European Neuroendocrine Tumor Society (ENETS) Center of Excellence, Department of Endocrinology, Diabetes and Metabolism, University Hospital Essen, Germany. We enrolled adults aged 16–87 years with biochemically proven endogenous hyperinsulinemic hypoglycemia in the fasting state (i.e. neuroglycopenic symptoms in the fasting state with low plasma glucose, inappropriately high serum insulin and C-peptide concentrations, and a negative screening for sulfonylurea). All patients had histological evidence of a differentiated neuroendocrine tumor, obtained from either a surgical specimen or a biopsy of a metastasis. Patients with poorly differentiated neuroendocrine carcinoma or any other malignant disease were excluded. Also excluded were patients who had undergone bariatric surgery, those with severe liver or kidney dysfunction, and those currently taking GLP-1-containing medications. Patients were treated at our center between June 2010 and July 2024 with all records and follow-up in our endocrine tumor center. All patients were presented to a multidisciplinary tumor board at diagnosis and at key decision points during the course of treatment, including initiation of therapy, evaluation of treatment response, and consideration of alternative management strategies. Tumor response was assessed according to the Response Evaluation Criteria in Solid Tumors (RECIST) version 1.1.

### Diagnostic procedures and laboratory methods

In cases of suspected insulinoma, dynamic endocrine testing was routinely performed in accordance with our institutional standard operating procedures. This diagnostic approach includes both a 5-hour oral glucose tolerance test (OGTT) and a supervised 72-hour fasting test, conducted sequentially where appropriate. In patients with spontaneous hypoglycemia with markedly elevated insulin and C-peptide levels at baseline, dynamic testing was omitted as biochemical confirmation of insulinoma was considered sufficient. To ensure test integrity and exclude the intake of hypoglycemia-inducing agents, beta-hydroxybutyrate and oral antidiabetic drug levels including sulfonylureas and glinides and toxicology screening were routinely measured. This standardized testing strategy ensures assessment of both fasting and reactive hypoglycemia, supporting the accurate biochemical diagnosis of insulinoma.

All assays were performed in the Central Laboratory (University Medicine Essen) under standardized pre-analytical conditions and internal quality control. Insulin was measured by a two-step solid-phase chemiluminescence immunometric assay on the Immulite 2000 XPi (Siemens Healthineers; fasting reference 3.6-29.1 µU/ml). C-peptide was measured by competitive chemiluminescence immunoassay on the same platform (reference 0.9-6.9 ng/mL). Glucose was measured by a hexokinase-based enzymatic assay with photometric detection at 340 nm on the Atellica CH (Siemens Healthineers; fasting reference 74–109 mg/dl). Proinsulin (total) was quantified by enzyme immunoassay (reference 3.3-28.0 pmol/L). Analytical accuracy was ensured through strict adherence to standardized protocols and regular calibration of laboratory equipment.

The histopathological data for each patient were obtained from the reports of the Department of Pathology at our ENETS Center. The reports included information on morphology, grading, and immunohistochemical staining for chromogranin A (CgA), synaptophysin, and insulin. The grading was based on the World Health Organization (WHO) Classification of Tumors of the Digestive System, 5th Edition (2019): Grade (G) 1 (Ki-67 index <3%), G2 (Ki-67 index 3–20%), and G3 (Ki-67 index >20%), with poorly differentiated neuroendocrine carcinomas classified separately (6). For tumor staging, the TNM classification system (tumor, lymph nodes, metastases) of the Union for International Cancer Control/American Joint Committee on Cancer (UICC/AJCC) for malignant tumors, 8th edition (2016), was used. All patients underwent cross-sectional imaging (computed tomography [CT] or magnetic resonance imaging [MRI]) in accordance with standard clinical practice.

Insulinomas were classified as non-metastatic if hypoglycemic symptoms were completely and permanently resolved following the removal of a localized pNET and if there was no evidence of metastasis upon histological examination. Metastatic insulinomas were defined as those cases in which hypoglycemic episodes persisted, or recurred despite initial treatment, combined with histological evidence of advanced pNETs (e.g. positive regional lymph nodes or distant metastases). Patients with inconclusive clinical findings, such as hypoglycemia without a visually apparent NET or a visually apparent NET without concomitant hypoglycemia, were not included in the study.

### Statistics

Data are reported as number of patients (percentage) for categorical variables. Continuous variables are reported as median with interquartile range (IQR) or range, as appropriate. Time-to-event outcomes are reported as median survival with 95% confidence intervals (CI). Overall survival (OS) was measured from initial NET diagnosis to death from any cause. Insulinoma-specific survival (ISS) was measured from the date of insulinoma diagnosis to insulinoma-related death; deaths from other causes were censored. Survival was estimated using the Kaplan-Meier method and compared using the log-rank test. Tests were two-tailed and results at p < 0.05 were considered statistically significant.

To predict metastasis, a multivariable logistic regression was performed with three routinely available predictors: tumor size (mm), fasting glucose (mg/dl) and the insulin-to-C-peptide ratio. Discriminative performance was summarized by the area under the receiver operating characteristic curve (AUC) with 95% confidence intervals (CI). Decision performance is reported as sensitivity, specificity, positive predictive value (PPV) and negative predictive value (NPV) at a pre-specified operating point (predicted probability 0.328). The final model was applied unchanged to an independent set of 24 published adult insulinoma cases that reported all predictors; variables were converted to the same units as in the cohort before prediction. External performance included AUC, sensitivity, specificity, PPV, NPV, and overall accuracy. This analysis examined applicability across heterogeneous reports and did not involve model refitting or threshold adjustment.

Decision curve analysis (DCA) was performed to evaluate the clinical net benefit of the prediction model across a range of threshold probabilities. The model was compared with default strategies assuming that either all patients or no patients had metastatic insulinoma.

We reported our results using the strengthening the reporting of observational studies (STROBE) guidelines for cohort studies. All statistical analyses were performed using R 4.5.2 (R Foundation for Statistical Computing, Vienna, Austria). Survival analyses were performed using the survival and survminer packages. ROC analyses, AUC values and 95% confidence intervals were calculated using the pROC package. Sensitivity, specificity, PPV and NPV were calculated from confusion matrices. Decision curve analysis was performed using the dcurves package. Figures were generated using ggplot2. The study was approved by the Ethics Committee of the Medical Faculty of the University of Duisburg-Essen (18-8367-BO).

## Results

### Patient characteristics

**Figure 1:**
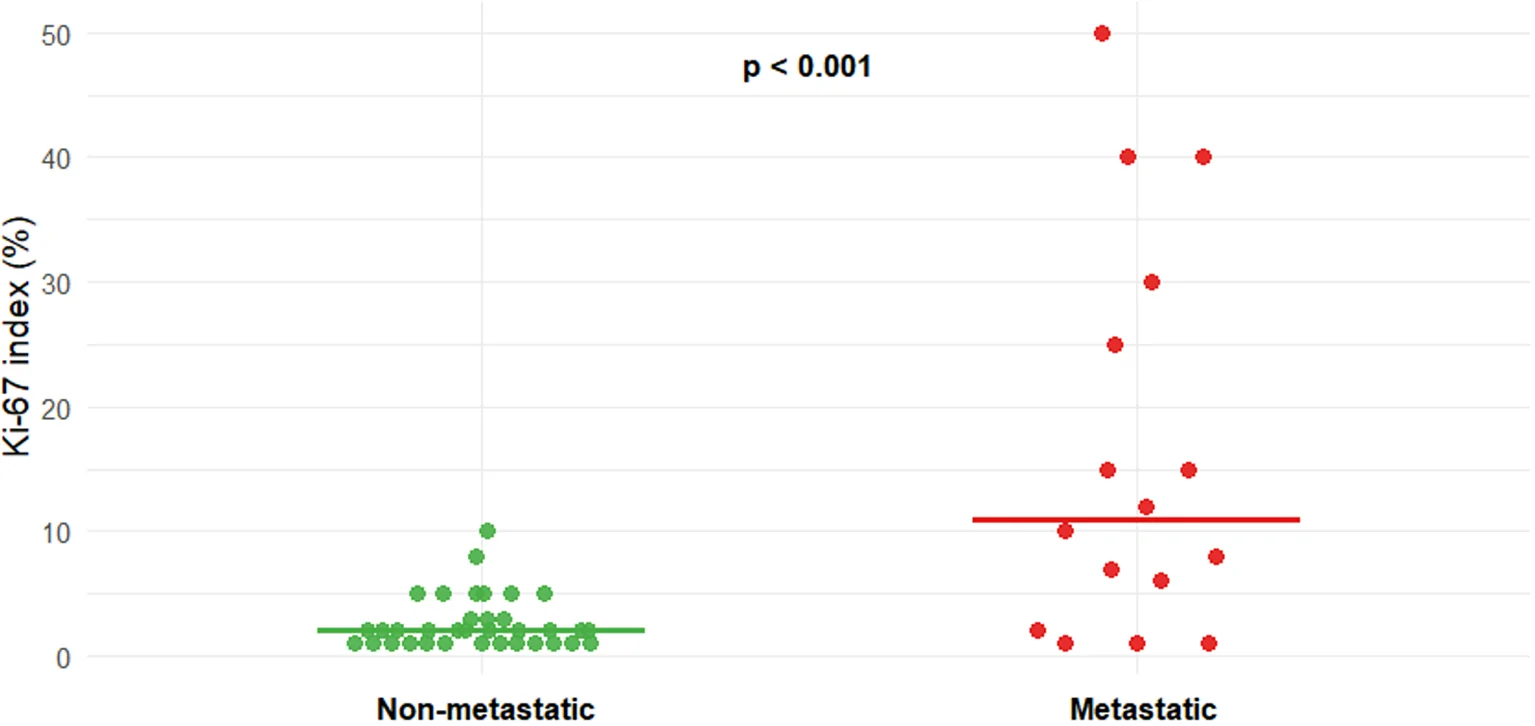
Ki-67-index in non-metastatic and metastatic insulinoma, horizontal lines indicate median values for each group (p<0.001).

**Figure 2:**
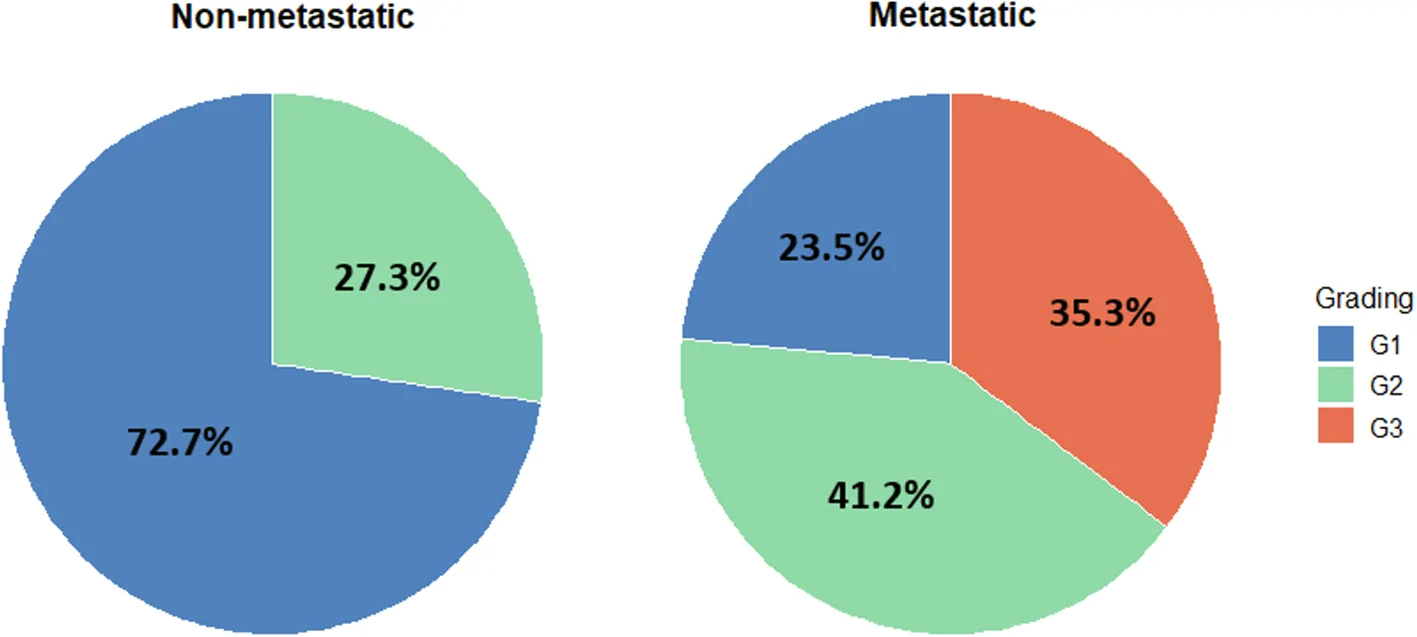
Distribution of grading in non-metastatic and metastatic insulinoma.

The study cohort included 64 patients with biochemically confirmed insulinoma, of whom 47 (73%) were classified as non-metastatic and 17 (27%) as aggressive based on the presence of metastases (**Table 1**). The sites of metastasis included the liver (n=15/17, 88%), lymph nodes (n=12/17, 71%), bone (n=5/17, 29%), peritoneum (n=2/17, 12%), duodenum (n=2/17, 12%), and others (n=2/17, 12%) (see the **Supplementary Appendix** for details). The median age at diagnosis differed significantly and was 47 years in the non-metastatic and 57 years in the metastatic subgroup (p = 0.018). Sex distribution differed between groups, with females predominating in non-metastatic cases (66%), while a higher proportion of metastatic cases were male (70.6%, p = 0.009). The Ki-67 index was significantly elevated in metastatic cases (**Figure 1**), with a median of 11% compared to 2% in non-metastatic tumors (p < 0.001). Accordingly, metastatic insulinomas were more frequently classified as G2 (n = 7) and G3 (n = 6), whereas none of the non-metastatic tumors were graded G3 (p < 0.001; **Figure 2**).

**Table 1:** Patient characteristics of the Essen insulinoma cohort, denominators vary due to missing data; available n is shown per variable.

Characteristic	Total cohort (n=64)	Non-metastatic (n=47)	Metastatic (n=17)	p-value
Median age, years (range)	49 (16–87)	47 (16–87)	57 (40-78)	<0.05*
Sex, n (%) Male Female	28 (44%) 36 (56%)	16 (34%) 31 (66%)	12 (71%) 5 (29%)	<0.05*
Ki-67-index, % (range)	2 (1-50)	2 (1-10)	11 (1-50)	<0.001***
Grading, n G1 G2 G3	28 16 6	24 9 0	4 7 6	<0.001***
Tumor Size, mm (range)	19 (5-110)	15 (5-65)	32 (20-110)	<0.001***
Deceased, n (%)	14 (22%)	3 (6.4%)	11 (64.7%)	
Insulinoma-related cause of death, n (%)	11 (17%)	0	11 (64.7%)	
Median insulinoma-specific survival, months [CI]	197 [90, 304]	not reached (94% alive at last follow-up)	51 [34, 68]	<0.001***
Time between symptom onset and diagnosis, months (range)	7.5 (0-368) (n=38)	8 (0-368) (n=33)	2 (0-18) (n=5)	
Primary diagnosis at our center, n (%)	36 (56%)	34 (72%)	2 (12%)	
Biochemical markers				
Insulin (µU/ml)	17	14	101	<0.001***
Glucose (mg/dl)	52	54	45	ns
C-Peptide (ng/ml)	2.5	2.2	3.9	<0.05*
Insulin/Glucose ratio	0.3	0.2	1.8	<0.001***
Insulin/C-Peptide ratio	6.1	5	20	<0.001***
Proinsulin (pmol/L)	4.2	3.5	33	
Proinsulin/Insulin ratio	0.5	0.4	2.5	
HOMA-IR	1.4	1	2.8	
β-hydroxybutyrate (µmol/L)	322	418	60	
Median glucose during OGTT				
0 min	66	66	–	
60 min	126	126	–	
120 min	111	111	–	
180 min	78	78	–	
240 min	60	60	–	
300 min	52	52	–	
Median fasting test duration, hours	23.5 (n=35)	24 (n=30)	2 (n=5)	< 0.05

*p < 0.05; ***p < 0.001.

The median follow-up period was 23 months (range 1-154). At the time of data cut-off, 14 of 64 patients (22%) had died. All insulinoma-related deaths (n = 11) occurred in the metastatic group (p<0.001), three deaths in the non-metastatic insulinoma group were attributed to postoperative complications following umbilical hernia repair, septic shock in the setting of dialysis-dependent chronic kidney disease and right heart failure due to a pulmonary carcinoid syndrome.

Among patients with non-metastatic insulinomas, 36 (77%) received specialized endocrine care at our unit within one month of diagnosis, either because the diagnosis was established at our institution or because they were promptly referred from external centers. In contrast, only 4 of 17 patients (23%) with metastatic insulinomas were seen within this timeframe. For the remaining patients, the median interval from diagnosis to first presentation at our department was seven months in the non-metastatic group and 19 months in the metastatic group. This marked delay in the evaluation of metastatic insulinomas was largely attributable to initial diagnostic and therapeutic management at external hospitals, with referrals typically occurring only after clinical deterioration, refractory hypoglycemia, or recurrence of hypoglycemic episodes. Six of the 64 patients (10%) had multiple endocrine neoplasia type 1 (MEN1), and in all of these cases the insulinoma was non-metastatic. Patients with MEN1 were significantly younger at the time of insulinoma diagnosis (median age 31 years) compared with the rest of the cohort (p = 0.014). In addition, eight patients (13%) had a prior diagnosis of a non-functioning pNET; in all of these cases the tumor was metastatic, and the development of insulin excess represented part of the pNET’s aggressive transformation. Local tumor recurrence was documented in four patients (6%), all of whom had an initially non-metastatic insulinoma.

### Diagnostics

In total, 36 of 64 patients (56%) received their initial insulinoma diagnosis at our institution, while 28 patients (44%) were diagnosed externally and subsequently referred for specialized management. This distribution differed markedly between groups, with 72% of non-metastatic insulinomas (34/47) diagnosed internally, whereas metastatic cases were predominantly identified externally (15/17; 88%), with only two metastatic tumors (12%) first diagnosed at our center.

Patients with metastatic insulinomas showed significantly higher baseline insulin concentrations, with a median of 101 µU/ml compared to 14 µU/ml in non-metastatic cases (p <0.001).

C-peptide levels were also elevated in metastatic cases (median 3.9 vs. 2.2 ng/ml, p < 0.05).

Proinsulin concentrations were higher in the metastatic group (33 vs. 3.5 pmol/L) and the proinsulin-to-insulin ratio was increased (2.5 vs. 0.4). Due to limited data availability and small subgroup sizes in metastatic insulinomas, statistical comparisons were not performed for proinsulin-related measures, β-hydroxybutyrate, and HOMA-IR.

**Figure 3:**
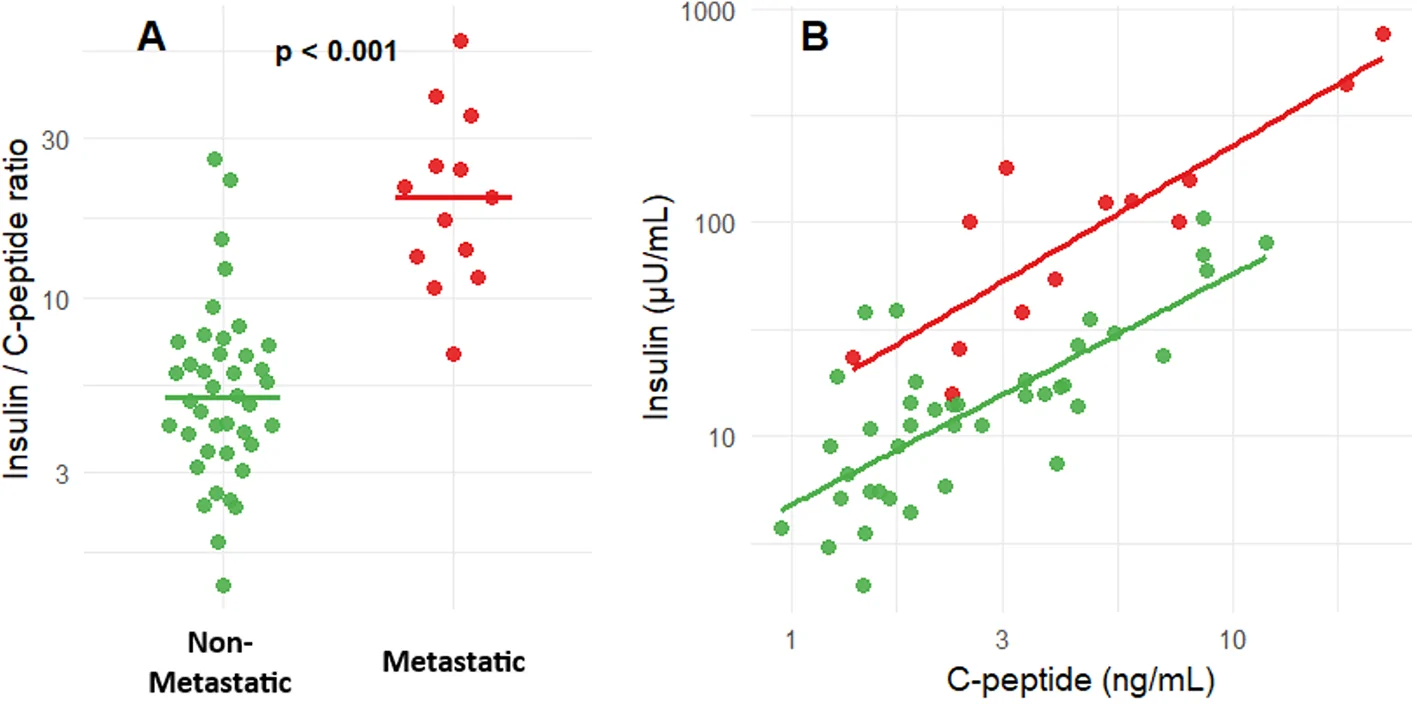
**(A)** Insulin-to-C-peptide ratios in non-metastatic and metastatic insulinomas. **(B)** Relationship between insulin and C-peptide concentrations in non-metastatic (green) and metastatic (red) insulinomas.

The insulin-to-glucose ratio was significantly higher in metastatic insulinomas (1.8 vs. 0.2, p < 0.001). Insulin elevation was therefore disproportionate relative to C-peptide, resulting in a significantly increased insulin-to-C-peptide ratio. Because both peptides are normally secreted in equimolar amounts, this disproportion suggests impaired prohormone processing. The markedly elevated proinsulin concentrations further support this interpretation, indicating a partial loss of β-cell differentiation and defective granule acidification (**Figure 3**).

Although glucose concentrations were lower in metastatic insulinomas (median 45 vs. 54 mg/dl), the difference was not statistically significant.

The HOMA-IR was higher in metastatic insulinomas, with a median of 2.8 compared to 1 in non-metastatic tumors, however, it was measured in only three metastatic insulinoma patients.

The 5-hour OGTT was performed in 26 patients (55%) with non-metastatic insulinomas. A fasting test was conducted in 43 patients of both groups (73%). Patients with metastatic insulinomas developed symptomatic hypoglycemia (n=5) significantly earlier, with a median duration of two hours compared to 24 hours in non-metastatic cases (p <0.05). A selective arterial calcium stimulation test (SACST) for tumor localization was performed in seven patients (11%) of which six had a non-metastatic and one a metastatic insulinoma.

### Tumor size

**Figure 4:**
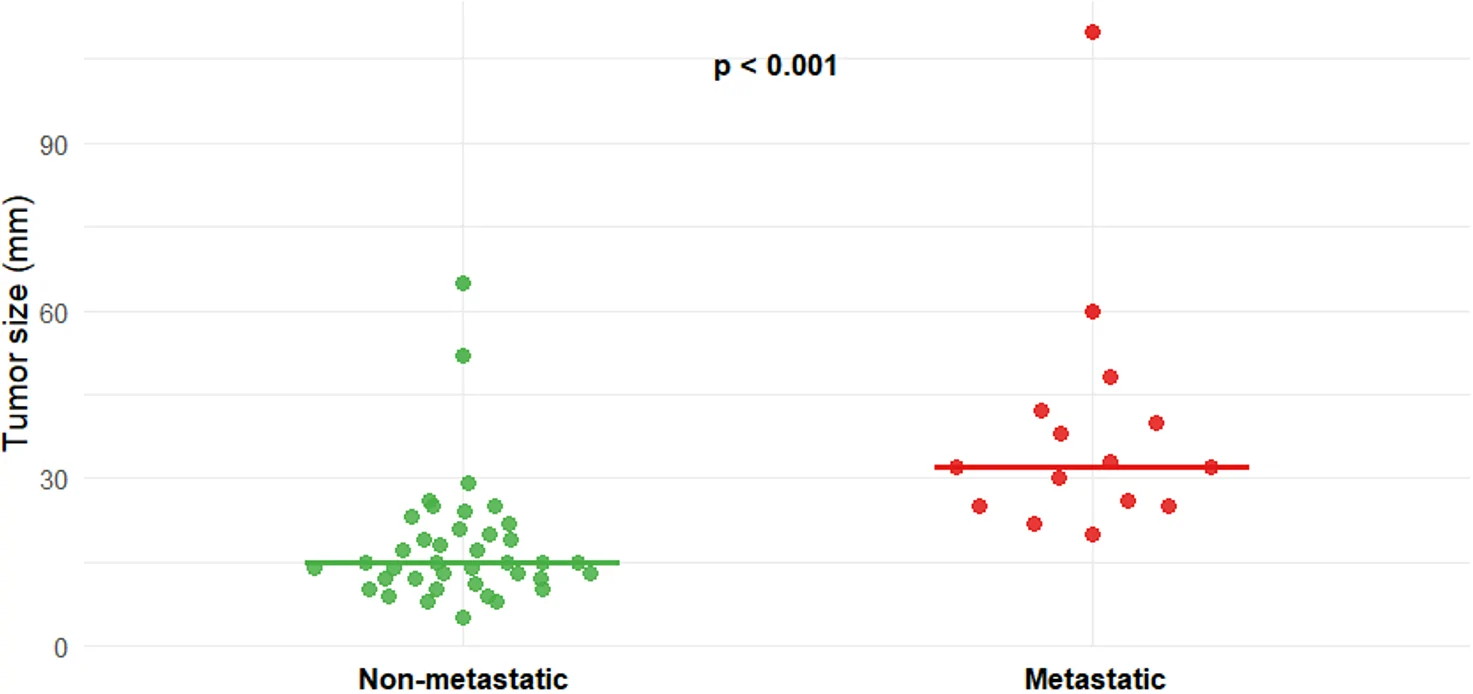
Tumor size in non-metastatic and metastatic insulinoma, horizontal lines indicate median values for each group (p<0.001).

Data on primary tumor size was available for 54 patients (39 non-metastatic and 15 metastatic insulinomas). The median tumor size in the entire cohort was 19 mm (IQR 13–27 mm). Metastatic insulinomas were significantly larger with a median size of 32 mm (IQR 25–42 mm) compared to 15 mm (IQR 12–21 mm) in non-metastatic cases (p<0.001, **Figure 4**). Receiver operating characteristic (ROC) analysis revealed that a primary tumor size >19 mm provided 100% sensitivity and 72% specificity in prediction of metastasis. A higher cut-off of >24 mm increased specificity to 85% with a slight reduction in sensitivity to 87%.

Chi-square test confirmed significant associations between tumor size cut-offs and malignancy (p < 0.001).

### Therapy

Therapeutic strategies differed significantly between non-metastatic and metastatic insulinoma cases (**Table 2**). Surgical resection was performed in 49 of 62 patients (79%). Eighty-seven percent of non-metastatic insulinomas (39/45) underwent surgery compared to 59% (10/17) of aggressive cases due to inoperability or metastatic spread at diagnosis. In these cases, metastatic disease was present at diagnosis and debulking surgery was performed to control severe hypoglycemia.

**Table 2:** Overview of therapies in the study cohort.

Therapy, n (%)	Total cohort	Non-metastatic	Metastatic
**Surgery**	49 (79%) (n=62)	39 (87%) (n=45)	10 (59%) (n=17)
**Targeted tumor therapy**	17 (29%) (n=59)	3 (7%) (n=43)	14 (88%) (n=16)
- Somatostatin analogs		3 (7%)	13 (81%)
- Everolimus		0	10 (63%)
- Sunitinib		0	5 (31%)
**Chemotherapy**	15 (23%) (n=64)	0 (n=47)	15 (88%) (n=17)
- Streptozotocin/5-FU		0	15 (88%)
- Temozolomide/Capecitabine		0	5 (30%)
- Cisplatin/Etoposide		0	1 (6%)
- Carboplatin/Etoposide		0	1 (6%)
**Interventional therapy**	14 (22%) (n=64)	2 (4%) (n=47)	12 (71%) (n=17)
- PRRT	9 (14%)	0	9 (53%)
- SIRT	7 (11%)	0	7 (41%)
- TACE	2 (3%)	0	2 (12%)
- RFA	2 (3%)	1 (2%)	1 (6%)
- Alcohol ablation	2 (3%)	2 (4%)	0

PRRT, Peptide Receptor Radionuclide Therapy; SIRT, Selective Internal Radiation Therapy; TACE, Transarterial Chemoembolization; RFA, Radiofrequency Ablation; 5-FU, 5-Fluorouracil.

Bold entries indicate the main treatment categories.

Eighty-eight percent of patients with metastatic insulinoma received targeted systemic therapy, most commonly somatostatin analogues (81%), followed by everolimus (63%) and sunitinib (31%), aimed at controlling hormone hypersecretion and tumor progression. Cytoreductive chemotherapy was administered in 88% (15/17) of metastatic insulinoma, most commonly with streptozotocin and 5-fluorouracil (88%) and temozolomide/capecitabine (29%), reflecting current recommendations for advanced pNETs (1). Combination regimens of e.g. diazoxide and steroids were occasionally necessary to manage therapy-resistant hypoglycemia in patients with high tumor burden.

In the non-metastatic insulinoma group, 3 of 47 patients required long-term medication. Among these, one received combined endoscopic and alcohol ablation, and another was treated with alcohol ablation alone. Local ablative therapy was chosen in these cases due to advanced age, comorbidities and patient preference. Despite the interventions, these patients ultimately required medical therapy with diazoxide, somatostatin analogs or corticosteroids to control hypoglycemia.

Interventional loco-regional treatments were predominantly used in the metastatic group. Twelve out of 17 patients (71%) with metastatic insulinomas underwent at least one interventional procedure, including peptide receptor radionuclide therapy (PRRT, 53%), selective internal radiation therapy (SIRT, 41%), transarterial chemoembolization (TACE, 12%) and in rare cases, ablation.

### Survival outcomes

The median OS for the entire cohort was 149 months (95% CI 61.8-not reached). During follow-up, 14 of 64 patients (22%) died.

**Figure 5:**
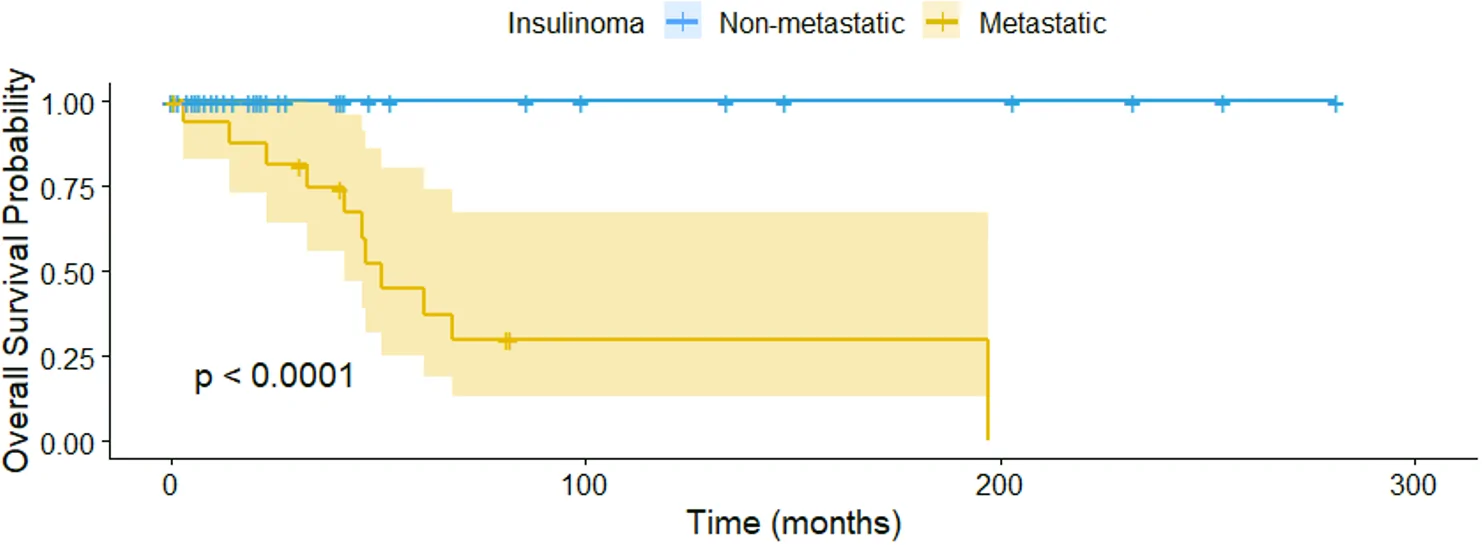
Kaplan-Meier estimate of insulinoma-specific survival in patients with non-metastatic (blue) and metastatic (yellow) insulinoma; non-insulinoma-related deaths were censored (n = 61).

The median insulinoma-specific survival (ISS) for the total cohort was 197 months (95% CI 89.7-304.3). Patients with non-metastatic insulinoma did not reach median ISS, and 94% were alive at last follow-up, with no insulinoma-related deaths observed. In contrast, metastatic insulinomas were associated with a median ISS of 51 months (95% CI 34.1-67.9; p < 0.001; **Figure 5**). The 5-year ISS rate was 38.5% in the metastatic cohort compared with 94% in the non-metastatic insulinoma group. When stratified by tumor size, patients with tumors >19 mm had a significantly shorter median ISS (61 months, 95% CI 33.7-88.2) than those with tumors ≤19 mm, for whom no insulinoma-related deaths occurred (p < 0.001). The corresponding 5-year ISS rate for tumors >19 mm was 49.2%. Similarly, patients with an insulin-to-C-peptide ratio >10.08 had a median ISS of 68 months (95% CI 36.5-99.4) and a 5-year ISS rate of 51.6%, whereas median ISS was not reached in patients below this threshold (p = 0.004).

### Predictive model for metastasis

We developed a multivariate logistic regression model to predict metastasis in insulinoma patients using three variables: basal insulin-to-C-peptide ratio, tumor size and fasting glucose. The insulin-to-C-peptide ratio emerged as an independent predictor (OR = 1.295; 95% CI, 1.06-1.68; p = 0.013), while tumor size (OR = 1.068; 95% CI, 0.99-1.15; p = 0.076) and fasting glucose (OR = 0.939; 95% CI, 0.88-1.00; p = 0.052) did not reach statistical significance individually but improved the overall model fit significantly (p = 0.019 for tumor size and p = 0.021 for glucose).

**Figure 6:**
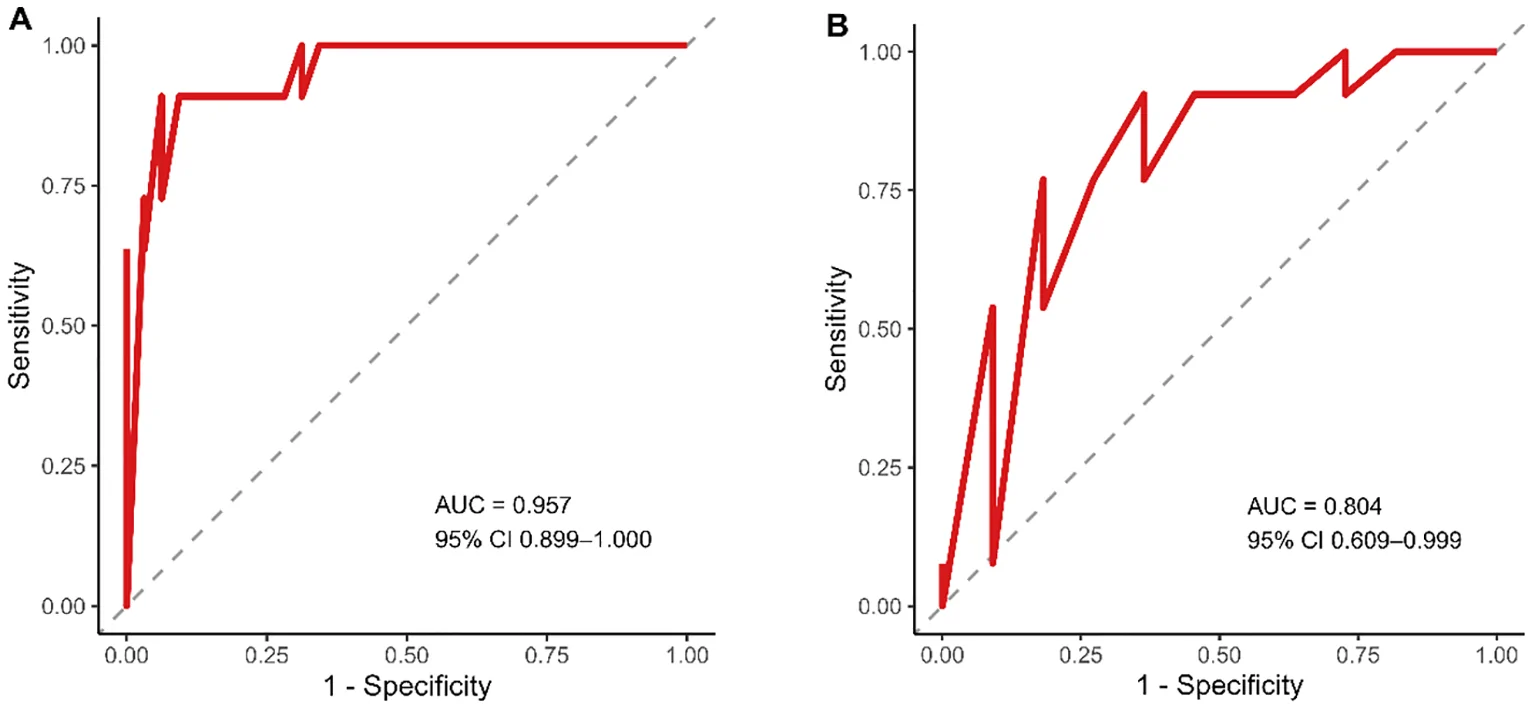
Performance of the insulinoma prediction model. **(A)** Receiver operating characteristic (ROC) curve of the derivation cohort demonstrating excellent discrimination for metastatic insulinoma (AUC 0.957). **(B)** ROC curve of the external validation cohort demonstrating good discrimination for metastatic insulinoma (AUC 0.804).

**Figure 7:**
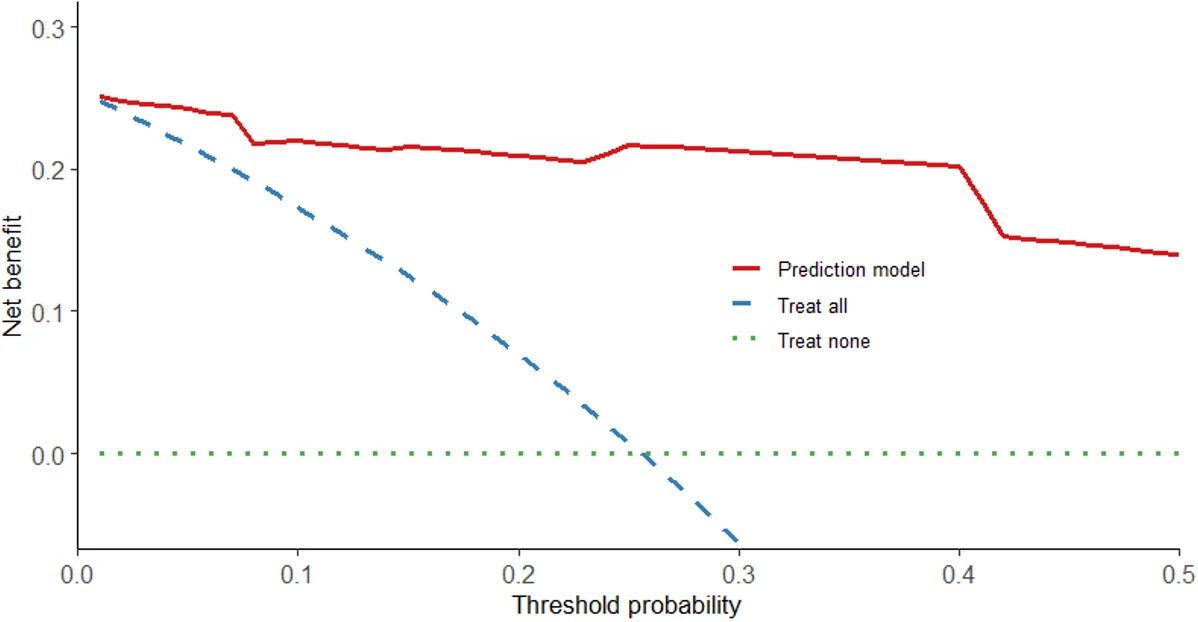
Decision curve analysis of the insulinoma prediction model. The prediction model provides a higher net benefit than the default strategies of treating all patients (“Treat all”) or no patients (“Treat none”) as having metastatic insulinoma across clinically relevant threshold probabilities.

The final model demonstrated excellent discriminatory performance with an AUC of 0.957 (95% CI, 0.899-1.000) and explained 75.6% of the variance (Nagelkerke R² = 0.756). The classification threshold was derived in the derivation cohort by maximizing the Youden index (probability cutoff 0.328). At this operating point, the model achieved a sensitivity of 90.9% and a specificity of 93.8% (**Figure 6A**). In the external validation cohort, the model demonstrated good discrimination with an AUC of 0.804 (95% CI, 0.609-0.999; **Figure 6B**). Decision curve analysis demonstrated a higher net benefit of the prediction model than default strategies assuming that all or no patients had metastatic insulinoma across clinically relevant threshold probabilities (**Figure 7**).

### External assessment

To evaluate the external validity of our predictive model, we applied it to a set of 24 published insulinoma cases for which sufficient clinical detail was available (**Table 3**). Initially, we used the predefined binary threshold (predicted probability ≥ 0.328) to distinguish between metastatic and non-metastatic tumors. Based on this cutoff, the model correctly classified 18 out of 24 cases (75%), with a sensitivity of 76.9%, specificity of 72.7%, PPV of 76.9% and NPV of 72.7%. This included 10 of 13 metastatic and eight of 11 non-metastatic insulinomas. Three metastatic cases were misclassified as non-metastatic and three non-metastatic cases were misclassified as metastatic.

**Table 3:** Cross-cohort application of the metastasis prediction model to 24 published cases of insulinoma.

ID	Tumor size (mm)	Glucose (mg/dl)	Insulin (µU/ml)	C-peptide (ng/ml)	Clinical classification	Model risk	Model risk prediction	Reference
1	16	45	8.3	2.4	Non-Metastatic	2.4%	Low	Ataallah (7)
2	8	57	5.3	7.5	Non-Metastatic	0.3%	Low	Kishi (8)
3	15	46	70	6.8	Non-Metastatic	11.2%	Medium	Tarchouli (9)
4	20	39	65	5	Non-Metastatic	35.3%	Medium	Tarchouli (9)
5	32	56	50	3.8	Non-Metastatic	29.9%	Medium	Alkhamisy (10)
6	130	23	34	3.9	Non-Metastatic	99.8%	High	Aslam (11)
7	15	50	40	4.8	Non-Metastatic	5.6%	Medium	Alobaydun (12)
8	41	29	18	1.3	Non-Metastatic	83.3%	High	Williams (13)
9	16	38	20	5	Non-Metastatic	4.2%	Medium	Ben Salem (14)
10	16	57	8.2	1.5	Non-Metastatic	1.9%	Low	Suminaga (15)
11	10	30	7.3	2	Non-Metastatic	4.3%	Medium	Matsumoto (16)
12	22	60	70	5.1	Metastatic	16.7%	Medium	Kishore (17)
13	150	45	17	4.1	Metastatic	99.4%	High	Ueda (18)
14	50	25.2	32	5.6	Metastatic	58.1%	High	Yu (19)
15	18	23.4	81	3.9	Metastatic	90.6%	High	Yu (19)
16	58	28.8	63	5.2	Metastatic	90.7%	High	Yu (19)
17	9	18.0	300	7.9	Metastatic	99.8%	High	Yu (19)
18	15	41.4	29	2.7	Metastatic	15.9%	Medium	Yu (19)
19	12	27.0	42	2.5	Metastatic	64.9%	High	Yu (19)
20	10	52	5.2	0.6	Metastatic	3.9%	Medium	Yu (19)
21	60	29	72	7.3	Metastatic	86%	High	Yu (19)
22	170	46	27	5.7	Metastatic	99.9%	High	Boateng (20)
23	58	25	38	6.7	Metastatic	70.1%	High	Lee (21)
24	43	40	89	4.2	Metastatic	95%	High	Ciacciarelli (22)

Model input variables were derived from published values.

**Figure 8:**
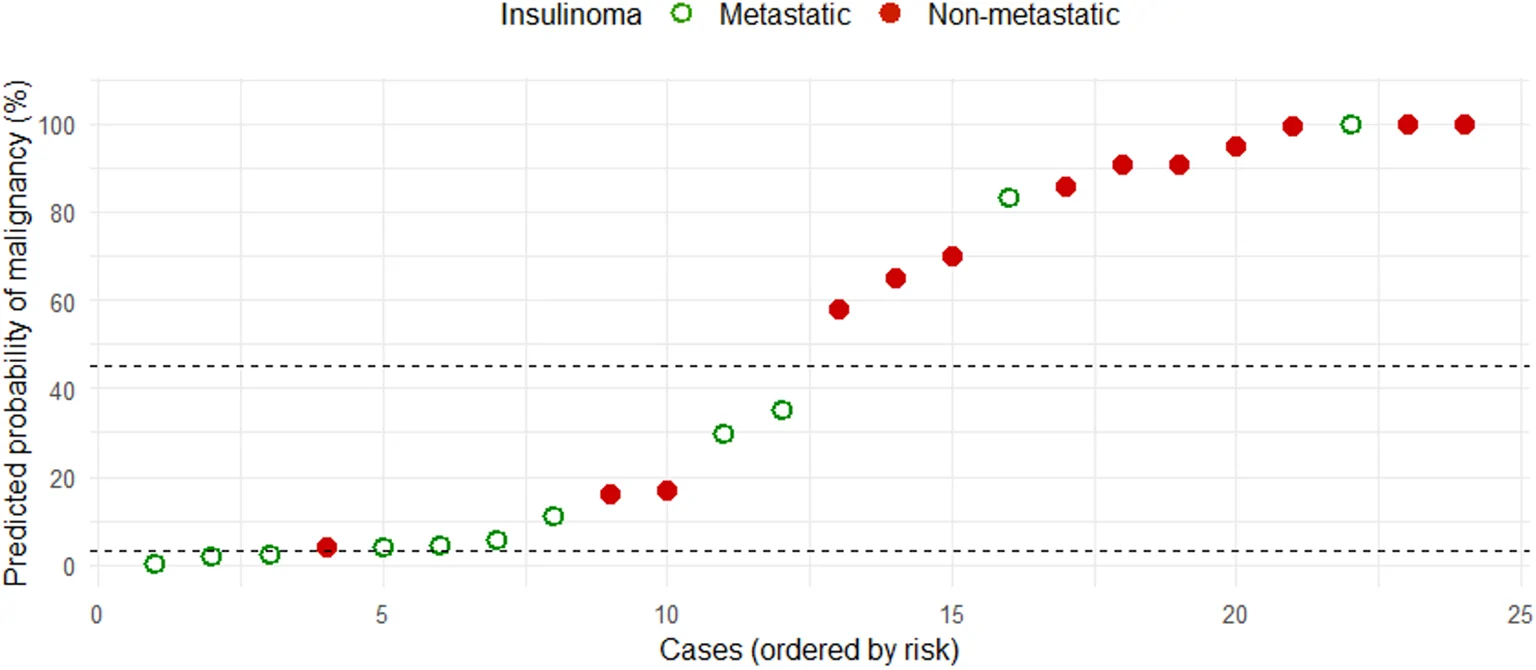
External assessment of the prediction model in 24 insulinoma cases; dashed lines at 3% and 45% define low-, medium- and high-risk groups.

To improve interpretability and clinical utility, we implemented a three-tiered risk stratification (low risk < 3%, medium risk 3-45%, high risk ≥ 45%, **Figure 8**). In this classification, no metastatic tumors were assigned to the low-risk group (NPV = 100%), ten of 13 metastatic tumors were classified in the high-risk group and three metastatic tumors fell into the medium-risk group. Among the 11 non-metastatic tumors, three were classified as low risk, six as medium risk and two as high risk. This yielded a sensitivity of 100% and an NPV of 100% for the low-risk group, while the high-risk group was predominantly metastatic (10/12 cases, PPV 83.3%).

One metastatic tumor (ID 20) with extensive disease was assigned only a 3.9% predicted risk, highlighting that rare misclassifications can occur in the lower range of the model’s output. Conversely, two non-metastatic tumors with very large primaries (ID 6: 130mm, ID 8: 41 mm) were categorized as high risk, reflecting the weight of tumor size in the prediction model.

**Figure 9:**
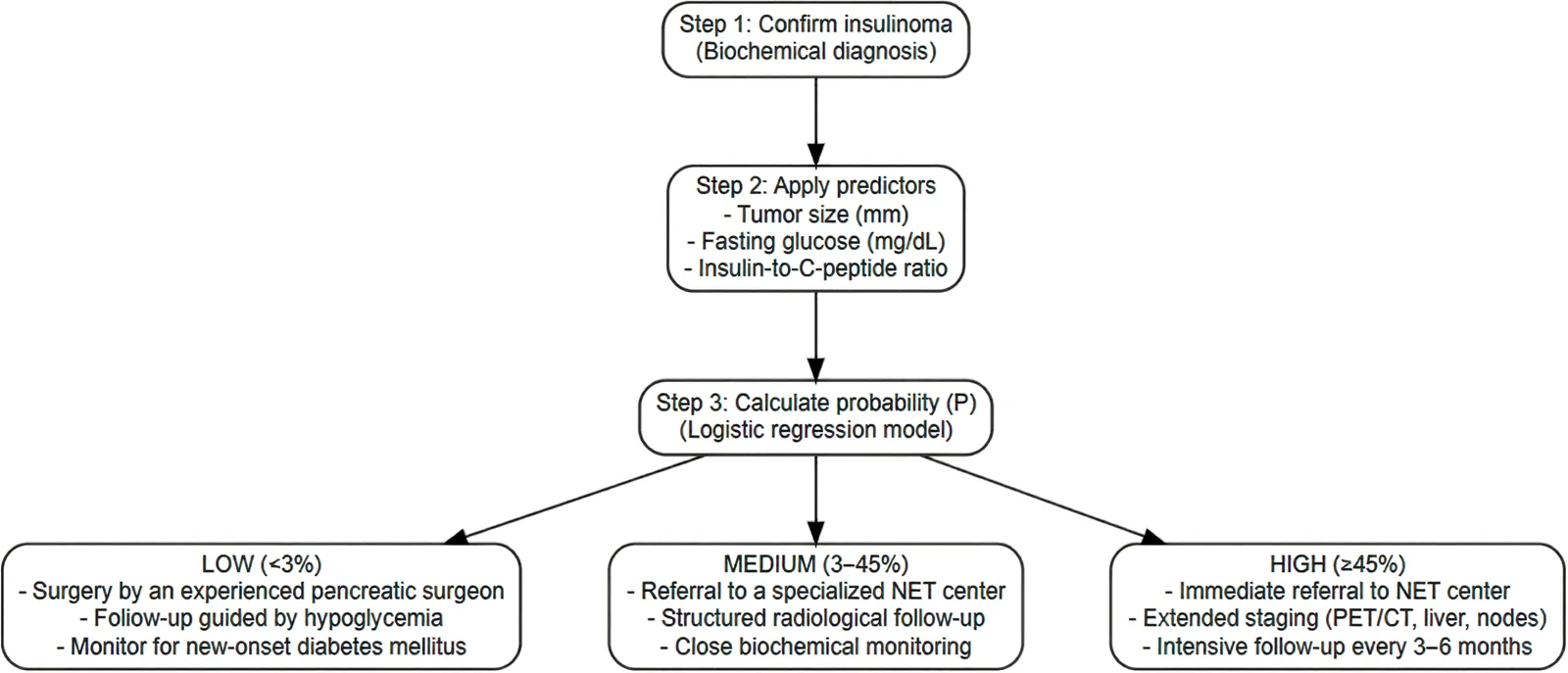
Clinical application of the risk model.

Overall, this external assessment supports the model’s applicability while emphasizing the importance of clinical judgment in interpreting model predictions, even when applied to heterogeneous real-world datasets with sometimes atypical behavior. The model-based stratification may guide the intensity of staging, treatment planning and follow-up in insulinoma patients (**Figure 9**).

## Discussion

Early differentiation of aggressive from non-metastatic insulinomas remains a clinical challenge due to overlapping biochemical and clinical characteristics and challenges in diagnosing insulinoma (23, 24). Our study addresses this by proposing a practical metastasis prediction model using tumor size, fasting glucose and the insulin-to-C-peptide ratio, three parameters readily available in standard clinical practice. To our knowledge, this represents the first model to stratify metastasis risk in patients with insulinoma.

Metastatic insulinomas accounted for 27% of cases, exceeding reported rates (4-16%), likely reflecting the tertiary referral nature of our center (3, 5, 23, 25, 26).

### Patient characteristics and diagnostics

The overall cohort showed a slight female predominance (56% female vs. 44% male), consistent with the literature (26). However, among metastatic insulinomas, males were significantly more predominant (71% vs. 29%, p < 0.05). This has also been observed in prior studies (27), suggesting potential sex-related differences in tumor biology.

Analysis of the cohort demonstrates that metastatic insulinomas are larger and more proliferative, exhibiting significantly higher Ki-67 indices. Clinically, they present earlier in the disease course (median 2 months in metastatic vs. 8 months in non-metastatic tumors), likely owing to the more rapid onset of severe neuroglycopenic symptoms. Biochemically, metastatic insulinomas demonstrated significantly elevated insulin, C-peptide and proinsulin concentrations at baseline. This was associated with insulin resistance, possibly due to chronic receptor downregulation and metabolic adaptation, representing a compensatory mechanism in aggressive insulin-secreting tumors (28, 29). We observed an elevated proinsulin-to-insulin ratio in metastatic insulinomas, suggesting impaired prohormone processing and reduced β-cell maturity. In normal physiology, pancreatic β-cells efficiently convert proinsulin to insulin within acidified secretory granules (30). A high ratio indicates a shift towards less differentiated cellular function, where this conversion becomes inefficient. Recent molecular studies support this finding, showing that metastatic insulinomas may lose typical β-cell characteristics and instead express markers associated with α-cell-like or progenitor states (29). This cellular dedifferentiation may promote their more aggressive behavior and underlines the potential value of molecular profiling in future risk stratification. Supporting this concept, previous case reports have described metastatic insulinomas with hyperproinsulinemia and loss of vacuolar-type H^+^-ATPase (V-ATPase) expression, an enzyme required for granule acidification and prohormone conversion (30). Impaired V-ATPase function and reduced activity of prohormone convertases PC1/3 and PC2 may cause the disproportionate proinsulin secretion observed in our cohort. These findings suggest that defective peptide processing is a possible feature of metastatic transformation with altered V-ATPase or convertase expression serving as immunohistochemical indicators of β-cell dedifferentiation (30).

As insulin bioactivity was not measured, the observed hypoglycemia may also result from high proinsulin concentrations. Although proinsulin has lower receptor affinity than insulin, excessive proinsulin secretion in metastatic insulinomas can contribute to hypoglycemia especially when proinsulin concentrations are very high or exceed those of insulin, as described in proinsulin-predominant tumors, so-called proinsulinomas (30, 31).

While fasting glucose alone was not significantly different between groups, its inclusion in the multivariate model improved predictive accuracy. This suggests that alterations in glucose regulation only become relevant when interpreted in the context of insulin and proinsulin dynamics.

This likely reflects that fasting glucose integrates insulin bioactivity and proinsulin processing defects. Subtle defects in peptide maturation may result in immunoreactive but biologically less potent insulin, influencing glycemia only when combined with high tumor burden or systemic adaptation. Therefore, the model’s combined use of biochemical and morphological parameters may reflect the multifactorial biology of metastasis rather than isolated metabolic markers.

### Therapy and survival

Therapeutic strategies in our cohort differed significantly between non-metastatic and metastatic insulinomas, reflecting the biological heterogeneity of the disease. Surgical resection remained the first-line treatment in 87% of non-metastatic cases but only in 59% of metastatic ones and was primarily directed at controlling rather than curing severe hypoglycemia. All metastatic insulinoma patients required antiproliferative systemic therapy, most commonly with somatostatin analogs, everolimus, sunitinib or chemotherapy consistent with ENETS recommendations for advanced pNETs. Interventional therapies such as PRRT, SIRT or TACE were frequently used (71%), underscoring the need for multimodal approaches in advanced disease. Recent data from Mayo Clinic sites with 57 metastatic insulinomas confirmed the heterogeneity of systemic therapy efficacy, with PRRT achieving the most consistent hypoglycemia control (93%), while temozolomide/capecitabine and everolimus provided both symptom relief and long median survival exceeding six years (32). Notably, metastatic insulinomas have been reported to exhibit a shift toward stronger somatostatin receptor subtype 2 (SSTR2) expression and simultaneous loss of glucagon-like peptide-1 (GLP1) receptor expression compared to non-metastatic insulinomas, supporting the rationale for PRRT or SSA as a targeted therapeutic option in this subset (1, 2).

In contrast, non-metastatic insulinomas were largely cured surgically, with only a small subgroup (n=4) requiring pharmacological or local ablative interventions due to comorbidities or patient preference. The optimal sequencing of multimodal therapies remains unresolved, underlining the need for an individualized, multidisciplinary approach to insulinoma management in the absence of standardized treatment algorithms for metastatic disease.

Survival outcomes in our cohort were similar to the literature, with non-metastatic insulinomas associated with excellent long-term survival (median not reached) and metastatic cases demonstrating poor prognosis (median OS 51 months) (33).

Although three deaths occurred in the non-metastatic insulinoma group, none were related to insulinoma or its treatment. These incidental deaths reflect comorbid conditions rather than tumor behavior and therefore do not affect conclusions regarding disease-specific prognosis. The difference in OS underlines the clinical relevance of early metastasis prediction. While previous studies report a median survival of approximately two years for metastatic insulinomas (24), our findings suggest improved outcomes under multimodal management at a specialized center.

The incidence of MEN1 in our cohort was 9.4%, which is higher than the reported rates of 5.7-7.6% in the literature (5, 25, 26). This may in part reflect surveillance bias, as systematic genetic testing and close clinical follow-up in our center may have increased MEN1 detection compared with less standardized approaches in other cohorts. All MEN1-associated insulinomas followed a non-metastatic course consistent with the literature.

### Predictive model and external assessment

The model demonstrated excellent discriminatory power (AUC = 0.957), with a 90% sensitivity and 93.8% specificity at the optimal threshold. External validation demonstrated good discrimination (AUC 0.804), supporting the applicability of the model in an independent cohort despite heterogeneous source data. Univariate ROC analysis confirmed tumor size as a strong predictor of metastasis, with a cut-off >19 mm yielding 100% sensitivity and >24 mm improving specificity to 84.6%. These thresholds may assist clinicians in assessing metastasis risk and in guiding decisions on early referral for multidisciplinary management. The longer referral delay observed in metastatic cases (19 vs. 7 months) further emphasizes the importance of early risk stratification to ensure timely access to specialized care and advanced therapeutic options.

When applied to an external dataset of 24 published insulinoma cases, for which clinical characteristics were available the model correctly classified 75% of cases using the pre-specified binary classification threshold. A probability-based stratification into low (<3%), medium (3-45%) and high (≥45%) risk categories further enhanced clinical interpretability. All metastatic tumors (13/13) were correctly identified as either medium or high risk (sensitivity 100%), while no metastatic cases were assigned to the low-risk group (NPV 100%). These findings support the potential clinical use of the model for triaging patients according to individualized metastasis risk, thereby defining the intensity of diagnostics, surveillance strategies and treatment planning.

According to current ENETS guidelines, patients with localized, low-grade, and non-metastatic insulinoma who undergo radical resection are considered cured and require only a single follow-up 3–6 months postoperatively. If hypoglycemic symptoms have resolved, no further routine follow-up is indicated, though patients should be advised to seek medical evaluation if symptoms recur. In contrast, patients with metastatic insulinoma require long-term follow-up comparable to that for other advanced or metastatic Pan-NETs, with clinical, biochemical, and imaging assessments every 3–12 months depending on tumor grade, burden and progression rate. As metachronous or shifting hormonal syndromes can develop over time, continued clinical observation and biochemical reassessment remain essential (1). Importantly, we observed functional transformation in eight patients whose tumors initially presented as non-functioning pNETs. All of these cases were metastatic, highlighting tumor plasticity.

Our model-based stratification complements this approach by identifying patients at low risk (<3%), who can safely follow standard minimal surveillance, in comparison to those at medium- or high-risk (≥3%), in whom structured radiologic and biochemical monitoring may be needed.

### Limitations and future directions

Limitations of our study include its small sample size (n= 64) due to the rarity of the disease. Hence, the proposed prediction model should be considered as exploratory. One metastatic case (ID 20) was classified in the medium-risk group, but with a very low predicted probability of malignancy (3.9%). Its small tumor size (10 mm) and borderline insulin-to-C-peptide ratio biased the model toward a non-metastatic prediction, despite documented malignant behavior. This highlights that rare metastatic insulinomas with small size may escape prediction models and require clinical attention regardless of model output. While the internal performance of our model was strong, external assessment was restricted to 24 case reports. Unusually large tumors classified as non-metastatic (e.g. 41 and 130 mm) in external case reports (11, 13) are very rare and may represent misclassification or reporting bias, which is a limitation of using heterogeneous published data for external assessment.

Hence, larger, multicenter prospective cohorts will be essential to confirm the generalizability of our proposed prediction model. Additionally, future studies incorporating molecular markers, imaging characteristics and longitudinal changes in biochemical parameters may add to further refine predictive accuracy.

## Conclusion

Metastatic insulinomas represent a rare but clinically highly significant subtype of insulin-secreting pancreatic neuroendocrine tumors. Patients are immediately threatened by recurrent and often severe hypoglycemia, which can lead to profound neuroglycopenia, neurological injury, and even life-threatening events if not promptly recognized. Compared with non-metastatic insulinomas, metastatic forms exhibit more aggressive biological behavior, including higher proliferative activity and larger tumor burden. This study introduces a practical prediction model for early metastatic risk stratification and delineates key clinical and biochemical differences between non-metastatic and metastatic disease. The combination of tumor size, fasting glucose, and the insulin-to-C-peptide ratio enables early identification of patients at elevated risk and supports more individualized diagnostic, therapeutic, and follow-up strategies. Early recognition and timely referral to specialized centers experienced in managing complex neuroendocrine tumors are essential to improve patient outcomes, and the proposed approach may enhance clinical decision-making while advancing personalized care for these rare endocrine tumors.

## Data Availability

The raw data supporting the conclusions of this article will be made available by the authors, without undue reservation.
