# Supplementary material for: Early prediction of metastatic insulinoma using tumor size and biochemical markers: a retrospective cohort study
Source: Front Endocrinol (Lausanne). 2026 Aug 25;17:1905732. doi: 10.3389/fendo.2026.1905732 (PMC13551242; doi:10.3389/fendo.2026.1905732)
Supplement: Supplementary file 1 [file DataSheet1.pdf]

Supplementary table

Localisation of metastases in patients with metastatic insulinoma.

Subject No.	Site(s) of metastasis*
1	1
2	3
3	1,2,3,4
4	1,2
5	1,2
6	1,2,6
7	1,2,6
8	1,2,5,6
9	1
10	1,2
11	1,2
12	1,7
13	2,7
14	1,2,6
15	1,2,6
16	1,2
17	1

* 1=liver, 2=lymph nodes, 3=duodenum, 4=peripancreatic tissue, 5=adrenal gland, 6=bone, 7=peritoneum
